# Adequate funding of comprehensive community‐based programs for key populations needed now more than ever to reach and sustain HIV targets

**DOI:** 10.1002/jia2.25967

**Published:** 2022-07-26

**Authors:** Meghan C. DiCarlo, Gina A. Dallabetta, Chris Akolo, Sergio Bautista‐Arredondo, H. Victor Digolo, Virginia A. Fonner, Grace Jill Kumwenda, Patrick Mbulaje, Peninah W. Mwangi, Navindra E. Persuad, Simon Sikwese, Tisha A. Wheeler, R. Cameron Wolf, Hally R. Mahler

**Affiliations:** ^1^ Global Health Population and Nutrition FHI 360 Washington DC USA; ^2^ HIV/TB Bill & Melinda Gates Foundation Washington DC USA; ^3^ Division of Health Economics and Health Systems Innovations National Institute of Public Health (INSP) Cuernavaca Mexico; ^4^ Men Against AIDS Group Organization (MAAYGO) Kisumu Kenya; ^5^ Pakachere Institute for Health and Development Communication Blantyre Malawi; ^6^ Centre for the Development of People Lilongwe Malawi; ^7^ Bar Hostess Empowerment and Support Program (BHESP) Nairobi Kenya; ^8^ Office of HIV/AIDS United States Agency for International Development (USAID) Washington DC USA

**Keywords:** Africa, community, differentiated care, HIV care continuum, key and vulnerable populations, structural interventions

## Abstract

**Introduction:**

Globally, over half of the estimated new HIV infections now occur among key populations, including men who have sex with men, sex workers, people who inject drugs, transgender individuals, and people in prisons and other closed settings, and their sexual partners. Reaching epidemic control will, for many countries, increasingly require intensified programming and targeted resource allocation to meet the needs of key populations and their sexual partners. However, insufficient funding, both in terms of overall amounts and the way the funding is spent, contributes to the systematic marginalization of key populations from needed HIV services.

**Discussion:**

The Joint United Nations Programme on HIV/AIDS (UNAIDS) has recently highlighted the urgent need to take action to end inequalities, including those faced by key populations, which have only been exacerbated by the COVID‐19 pandemic. To address these inequalities and improve health outcomes, key population programs must expand the use of a trusted access platform, scale up differentiated service delivery models tailored to the needs of key populations, rollout structural interventions and ensure service integration. These critical program elements are often considered “extras,” not necessities, and consequently costing studies of key population programs systematically underestimate the total and unitary costs of services for key populations. Findings from a recent costing study from the LINKAGES project suggest that adequate funding for these four program elements can yield benefits in program performance. Despite this and other evidence, the lack of data on the true costs of these elements and the costs of failing to provide them prevents sufficient investment in these critical elements.

**Conclusions:**

As nations strive to reach the 2030 UNAIDS goals, donors, governments and implementers should reconsider the true, but often hidden costs in future healthcare dollars and in lives if they fail to invest in the community‐based and community‐driven key population programs that address structural inequities. Supporting these efforts contributes to closing the remaining gaps in the 95‐95‐95 goals. The financial and opportunity cost of perpetuating inequities and missing those who must be reached in the last mile of HIV epidemic control must be considered.

## 1 INTRODUCTION

Globally, over half of the estimated new HIV infections now occur among key populations, including men who have sex with men (MSM), sex workers, people who inject drugs, transgender individuals, and people in prisons and other closed settings, and their sexual partners [1]. Individuals from key populations are more likely to lack access to prevention and treatment services, have undiagnosed HIV and fail to achieve viral suppression due to issues around stigma and discrimination, criminalization, violence and inadequate client‐centred service delivery approaches [2, 3].

To reach the Joint United Nations Programme on HIV/AIDS (UNAIDS) 95‐95‐95 treatment goals [4], we must address key population needs. For many countries meeting these needs will require intensified programming and targeted resource allocation, including programming addressing and counteracting the systematic marginalization faced by key populations.

Insufficient funding, both in terms of overall amounts and lack of funding prioritization for comprehensive key population services, contributes to this systematic marginalization. In the current context of flatlined HIV budgets with donors and governments [5, 6], key population programs remain grossly underfunded and disproportionately affected [7]. This precarious position is further exacerbated by uncertainties surrounding future HIV funding within the context of COVID‐19.

The contribution of key populations in generalized HIV epidemics has historically been underestimated, largely due to failing to consider differing transmission dynamics [8]. Evidence suggests that unmet prevention and treatment needs among key populations contribute the highest population‐level long‐term risk of onward transmission [9, 10]. Thus, allocating more resources towards filling the “prevention gaps” among key populations could produce the most cost‐effective programming [11, 12]. For example, a modelling study in Côte d'Ivoire suggests that HIV prevention and treatment services targeted to key populations could be the most cost‐effective way to improve national HIV control efforts by averting up to 30% of new infections [13]. Modelling from Zimbabwe shows that compared with a scenario in which the HIV epidemic continues unabated, and prevention, testing and treatment services remain constant, there would be 85% fewer new infections in 2030 had transmission through sex work been eliminated from 2010 onwards [14].

Despite evidence that increased investment in key population‐specific programming is needed for epidemic control, key elements of comprehensive, community‐based programming remain underfunded and underprioritized [7, 15]. Below, we outline core elements necessary for key population comprehensive services, discuss current spending patterns and costs, and provide recommendations to achieve increased investment.

## 2 DISCUSSION

### 2.1 The need to address inequalities

UNAIDS has recently highlighted the urgent need to end inequalities surrounding factors, such as health status, sexual orientation, drug use and gender identity [16]. Research has demonstrated that countries with legal protections in place for sexual minorities have lower HIV prevalence among key populations as compared to those without protections [17]. Data also support a link between experiencing human rights abuses and increased HIV prevalence [18]. These inequalities are particularly relevant within the context of COVID‐19, which disproportionately affects key and vulnerable populations [19]. According to UNAIDS, experience from pandemics and epidemics, such as COVID‐19, HIV and Ebola, has shown that key populations are more likely to experience food insecurity, face barriers to healthcare and suffer loss of livelihood, unemployment, homelessness and violence [20]. A failure to address these inequalities by providing dedicated funding will be a failure to reach HIV epidemic control.

### 2.2 Addressing inequalities and improving health outcomes among key populations is possible

To address inequalities and improve health outcomes among key populations, programs must expand the use of:

#### 2.2.1 A trusted access platform

Key population programs must be built with the foundation of a trusted access platform, a term to describe coordinated, client‐centred community‐ and clinic‐based efforts that work to establish trust, reach underserved key populations and encourage service utilization to improve health and wellbeing [21]. Many facility‐based models may fall short of addressing inequities and thus may fail to reach those in most need. Last‐mile approaches require deep, peer‐led reach into communities. At its core, a trusted access platform embraces the “nothing about us without us” philosophy as the platform works with key population communities across all program aspects [21]. Trusted access platforms may involve providing services directly through key population organizations or communities; increasing agency through solidarity and community mobilization; supporting community‐led monitoring; and increasing provider competencies to provide respectful services. Some services are examples of differentiated service delivery (DSD), described in more detail below. For example, in a review of Ashodaya Samithi, an organization run by and for sex workers in Mysore, India, sexually transmitted infection (STI) and HIV outcomes improved during a period of intensive outreach, condom distribution and clinic checkups. During this period, the proportion of sex workers seen for checkups with STI symptoms requiring treatment fell from 5% to less than 1%, and testing demonstrated a decline in recent HIV infections [22].

#### 2.2.2 DSD models tailored to the needs of key populations

DSD models are client‐centred approaches that adapt, simplify and tailor HIV services to better meet client needs and reduce unnecessary healthcare system burden [23]. These models are described in several global guideline documents relevant to key populations [24, 25, 26, 27]. Frequently, DSD models for key populations involve peers, often as educators, counsellors, mobilizers, navigators or simply as network members to leverage expert community knowledge, build trust and increase the uptake of community‐based services [28, 29, 30, 31]. For example, sites run by key populations, such as drop‐in centres (DICs) and community clinics, provide important treatment alternatives. From 2015 through 2019, the USAID Linkages across the Continuum of HIV Services for Key Populations Affected by HIV (LINKAGES) project supported civil society organization partners to establish 35 key population DICs in Kenya and 19 DICs in Malawi and gradually expand their service offerings, including provision of antiretroviral therapy (ART). This led to immediate improvements in enrolment and a six‐fold increase in ART initiations among individuals in key populations within the Kenya sites [32, 33]. Additionally, data from many LINKAGES countries have consistently showed higher case‐finding rates when using differentiated testing models [34]. In an analysis of the enhanced peer outreach approach (EPOA), a social network testing strategy, implemented by LINKAGES in Côte d'Ivoire among female sex workers (FSW), EPOA was associated with a higher HIV case‐finding rate (11% vs. 7%; *p* < 0.001), a higher proportion of linkage to treatment (96% vs. 71%; *p* <0.001) and a higher proportion of treatment initiation (79% vs. 73%; *p* = 0.107) [34].

DSD approaches can also improve access to prevention commodities, such as pre‐exposure prophylaxis (PrEP), and promote network‐based risk reduction [35, 36, 37]. For example, the LINKAGES project in Eswatini implemented community‐based PrEP initiation for key populations involving access to self‐testing, community PrEP ambassadors and PrEP refills through key population community centres, resulting in increased PrEP uptake [38]. In a small group of MSM reached through EPOA by the Meeting Targets and Maintaining Epidemic Control (EpiC) project in Lesotho, 68% of MSM who tested negative were counselled on PrEP, and of those 92% initiated PrEP [37].

Other successful DSD examples include peer‐led STI testing in Australia [28], delivery of HIV self‐test kits through men's social networks in Tanzania [30] and involving peers in HIV and harm reduction services for people who use drugs [28]. However, a lack of funding for community‐led demand generation for such services and lack of financial investment to adequately provide community‐based services—coupled with policy limitations—constrain DSD scale‐up [39].

#### 2.2.3 Structural interventions

Structural interventions seek to change aspects of the legal, political, socio‐economic and built environment that shape lives and ultimately impact health [40, 41]. Structural interventions that address the persistent stigma, discrimination, violence and human rights abuses faced by key populations lack political and financial investment, yet these interventions are often high priority needs among key populations [42]. Evidence from across sub‐Saharan Africa demonstrates that key structural elements, including discriminatory and punitive laws, human rights violations and stigma, work to increase HIV risk among key populations [43, 44]. Structural interventions can be a critical entry point for reaching key populations and can also involve increasing advocacy for funding priorities, thus impacting political environments that perpetuate inequities. For example, the Global Network of Sex Work Projects represents sex workers from across 60 countries and has stimulated global dialogue about funding priorities for sex worker health and wellbeing [45].

#### 2.2.4 Service integration

Funding towards service integration is needed to address inequities and serve holistic population needs. While structural interventions seek to address the upstream factors that cause inequities, service integration and person‐centred care acknowledge these inequities and develop service packages addressing their downstream impact [46]. For example, programs among key populations have successfully integrated HIV‐related services with mental healthcare [47], gender‐affirming care [48], harm reduction [29], STI and viral hepatitis diagnosis and treatment [49, 50, 51], tuberculosis [52], sexual and reproductive health [53] and services that address gender‐based violence [54]. As one specific example, through EpiC Kenya, 220 key population clients who were initially reached with services to address violence (e.g. community‐based violence response systems and paralegals) accessed HIV testing services during a 3‐month period [32].

While service integration itself may not be underfunded, funding for key population programming is often siloed into specific health areas, such as HIV, thus limiting programs’ ability to comprehensively address needs. Service integration also provides potential cost savings and efficiencies within programs.

### 2.3 Continuing to inadequately fund key population programs will prevent the global community from reaching the 95‐95‐95 treatment goals

Identifying individuals in key populations, understanding their needs and preferences, increasing the use of DSD models and service integration, and addressing the stigma, discrimination and violence faced by key populations undoubtedly entail upfront (and ongoing) costs not always present in facility‐based models. Furthermore, these elements, which provide a strong platform for prevention, are also absent in some community‐based models with no dedicated funding. We contend that investment in the core elements of comprehensive services is lacking both because there is insufficient information on their costs and because these components are often viewed as “extras,” not necessities. This perception is exacerbated by the fact that key population programs are often viewed as vertical “add‐ons” to existing programs, thus erroneously viewed as expensive and unnecessary. There is often a lack of guidance about the true costs of these elements and the costs of failing to provide them. Consequently, costing studies of key population programs frequently neglect to assess the full costs of providing robust services for key populations and systematically underestimate the total and unitary costs of services. More accurate cost data, as well as more data on the benefits of comprehensive programs, are needed.

**Figure 1:**
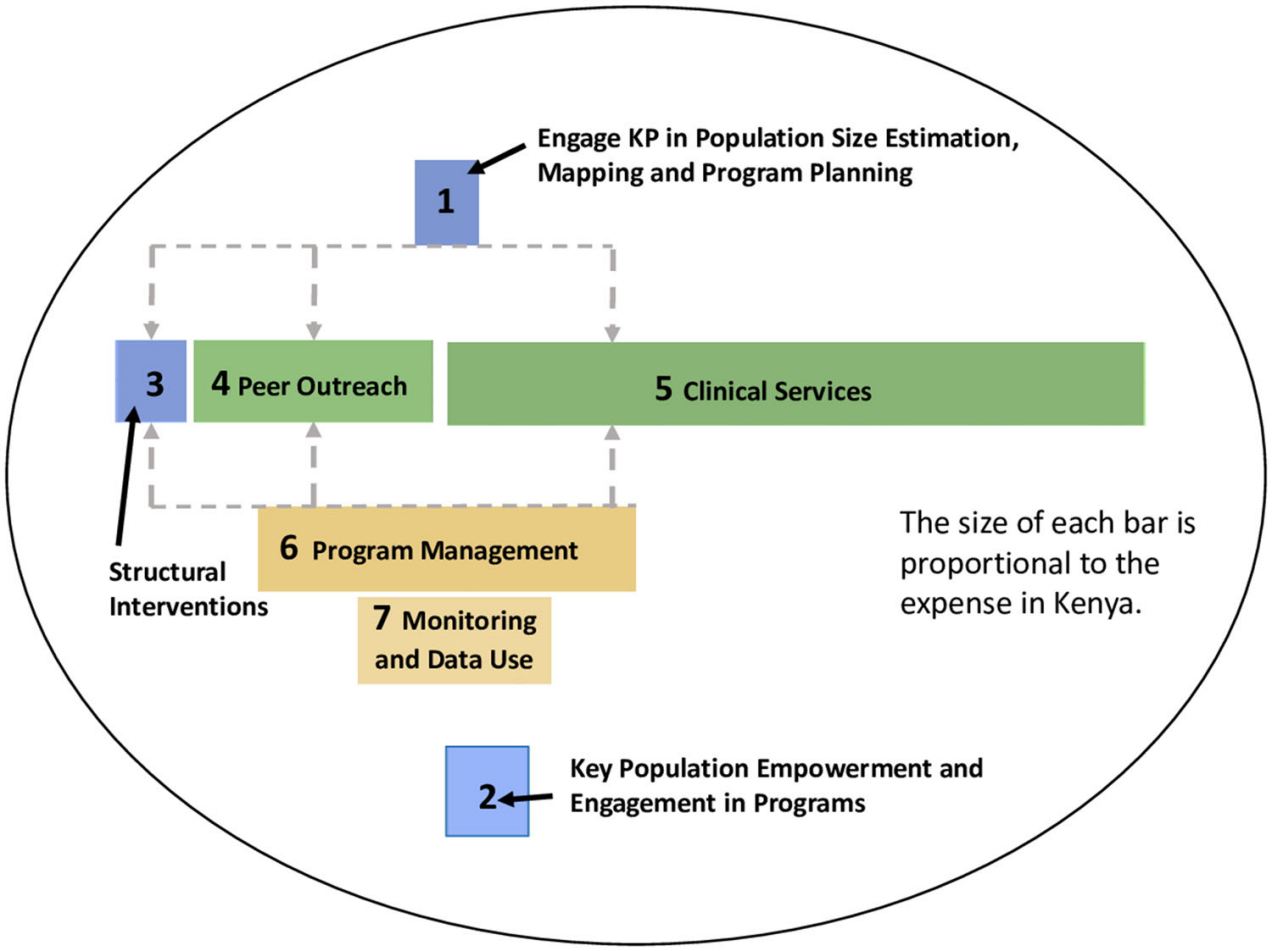
Relative weight of costs across program areas, LINKAGES Kenya. The figure represents results from a costing analysis of the LINKAGES program in Kenya, a comprehensive key population program implemented from 2015 to 2019. Within the figure, each program component is represented by a bar that is proportional to the size of the expense. Cross‐cutting elements are represented in blue, HIV cascade services in green and program management and monitoring in tan.

Although costing studies of comprehensive programs are scarce, several noteworthy examples exist. One study estimated costs within Avahan, a large‐scale, comprehensive HIV prevention program among FSW, high‐risk MSM and transgender individuals within India [55]. The program components, and their proportional costs were: peer outreach and services for STIs (22%), expertise enhancement (34%), community mobilization and enabling environment activities (7%) and program management (37%) [55]. A recent costing study from LINKAGES, which employed DSD models, key population engagement and peer cadres (e.g. peer educators and navigators) as program foundations, showed that clinical services comprised 40% of total cost in Kenya and 30% in Malawi, while peer outreach activities made up 13% of costs in Kenya and 16% in Malawi. Program management made up 22% of costs in Kenya and 19% in Malawi, comprising activities enabling efficient and high‐quality programming (e.g. supportive supervision, local organization capacity building, technical assistance and finance/operations support). Yet, even with these strong community components, the relative weight of costs across the LINKAGES program areas in Kenya shows that key population engagement/empowerment and structural interventions contributed the smallest proportion of costs (Figure 1). However, these critical elements in particular were underfunded, limiting the degree to which they could be implemented despite a recognized need from communities and implementers.

These results suggest that dedicating funding for critical elements of comprehensive services can generate program benefits with progressive decreases in unit costs following initial investments in community engagement, capacity building and program management.

However, as few costing examples exist, challenges remain in determining current funding levels allocated for key population services, and consequently, determining necessary funding levels to create and sustain comprehensive programs. The lack of quality costing data for these components could be contributing to a lack of prioritization among global funders and implementers. Additionally, given the emphasis within comprehensive HIV programs to address holistic needs, such as through structural interventions to address stigma and violence, such interventions can be undervalued when viewing their sector‐specific outcome potential in isolation (i.e. interventions to address violence viewed as having limited impact on HIV‐specific outcomes) [56]. Cross‐sectoral approaches and co‐financing models exist that could boost investment in structural interventions [57]. Additionally, expanding mechanisms to support community‐led organizations is essential [58].

## 3 CONCLUSIONS

As donors, governments and implementers strive to reach the 2030 UNAIDS goals, they should reconsider the true, but often hidden, costs not only in future healthcare dollars, but in lives of the failure to invest in comprehensive key population programs.

Supporting these efforts is an investment in closing remaining gaps in key population coverage with comprehensive programs and in the 95‐95‐95 treatment goals. This may include supporting the capacity of key population staff cadres in the health workforce (and having a management structure and human resources necessary to support such a cadre), moving beyond public sector facility models to integrate DSD models into national HIV strategies and policies, and adequately supporting interventions to address structural inequities. This becomes increasingly critical as epidemic “control” is achieved and where ongoing transmission will occur in marginalized populations, including key populations. Funding from governments to community‐based organizations should be established and expanded. Consider not only the costs but also the opportunity costs of perpetuating inequities and missing those who must be reached in the last mile of HIV epidemic control.

**Recommendations for increasing investment in comprehensive services for key populations**

**Program implementers and researchers**

- Collect data on cost and publish more data on cost/spending patterns for all comprehensive programming elements.
- Collect program data on structural interventions to show the value addition of these interventions, including their impact on HIV‐related service uptake.
- Challenge perception of critical elements of comprehensive programming as “extras” by evaluating and highlighting their critical role in creating effective, sustainable programs.
- In the absence of complete and comprehensive cost data, make assumptions using existing data to help inform how much these critical elements should cost.
- Develop advocacy plans to lobby and mobilize resources for structural interventions.

**Donors, funding agencies and governments**

- Prioritize comprehensive programming that includes community and DSD models.
- Provide more funding for structural interventions using multisectoral, co‐financing approaches.
- Provide guidance on budgeting for these critical elements.

## COMPETING INTERESTS

All authors declare no competing interests.

## AUTHORS’ CONTRIBUTIONS

MCD, GAD, SBA, RCW, TAW, HRM, NEP and CA developed the concept for this article. MCD drafted and edited the manuscript. VAF and MCD revised the manuscript. MCD, GAD, CA, SBA, HVD, GJK, PM, PWM, NEP, SES, TAW, RCW and HRM reviewed and commented upon the manuscript.

## FUNDING

The costing study from the LINKAGES program was funded by the Bill and Melinda Gates Foundation (Funding Grant Number: OPP1175038). LINKAGES was funded by the President's Emergency Plan for AIDS Relief (PEPFAR) through the United States Agency for International Development (Cooperative Agreement AID‐OAA‐A‐14‐00045).

## DISCLAIMER

The findings and conclusions in this article are those of the authors and do not necessarily represent the positions of the institutions with which they are affiliated or any of the funding agencies.

## Data Availability

Data sharing is not applicable to this article as no new data were created or analyzed in this study.
